# Short-Communication: Variable Expression of Clinical Symptoms and an Unexpected Finding of Vacuolar Myopathy Related to a Pathogenic Variant in the CACNA1S Gene in a Previous Case Report

**DOI:** 10.7759/cureus.23760

**Published:** 2022-04-02

**Authors:** Edmar O Benítez-Alonso, Juan C López-Hernández, Javier A Galnares-Olalde, Raúl E Alcalá, Edwin S Vargas-Cañas

**Affiliations:** 1 Neurogenetics, Instituto Nacional de Neurología y Neurocirugía Manuel Velasco Suárez, Mexico City, MEX; 2 Neuromuscular Diseases, Instituto Nacional de Neurología y Neurocirugía Manuel Velasco Suárez, Mexico City, MEX

**Keywords:** molecular analysis, vacuolar myopathy, pathogenic variant, hypokalemic periodic paralysis, cacna1s gene

## Abstract

Several clinical phenotypes have been described related to the *CACNA1S* gene (calcium channel voltage-dependent L-type alpha-1S subunit), such as autosomal dominant hypokalemic periodic paralysis 1 and autosomal dominant malignant hyperthermia susceptibility and are associated with autosomal dominant and recessive congenital myopathy. Recently, an interesting case of a 58-year-old male patient was published describing an unusual clinical presentation of hypokalemic periodic paralysis where a late-onset limb-girdle myopathy had developed 41 years after paralysis occurred when the patient was 11 years old. Muscle biopsy results were consistent with myopathic changes and revealed the presence of vacuoles, without inflammatory reaction. Later, molecular analysis revealed a pathogenic variant c.3716G>A (p.Arg1239His) in exon 30 of the *CACNA1S *gene. This technical report provides an extension of the molecular findings and evaluates the clinical and histopathological relationship previously published regarding this case.

## Introduction

Hypokalemic periodic paralysis (HypoPP) is a neuromuscular disorder caused by pathogenic variants in calcium or sodium channels with an autosomal dominant inheritance characterized by recurrent hypokalemia episodes and muscle weakness. The disease onset occurs in the first two decades of life and the most frequent symptom is paroxysmal muscle weakness secondary to strenuous physical activity or intake of carbohydrate-rich meals, followed by myopathy in limb-girdle pattern months or years after the onset of the first muscle weakness crisis [1,2].

We only identified one previous report of two individuals from the same family with the pathogenic variant p.Arg672Gly in the *SCN4A* gene and tubular aggregates in their muscular biopsies to date [3]. This work aims to review the literature focused on the most frequent mutations related to the *CACNA1S* gene (calcium channel voltage-dependent L-type alpha-1S subunit) and their clinical phenotypes.

## Technical report

**Figure 1:**
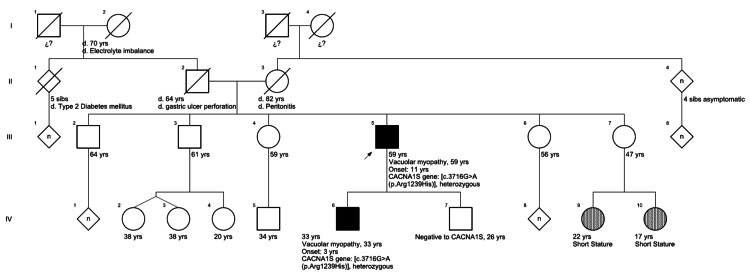
The family’s pedigree. The pathogenic variant c.3716G>A (p.Arg1239His) in exon 30 of the *CACNA1S* gene was detected in a heterozygous status in the proband (III-5) and his affected son (IV-6). His younger son (age 26) had a negative molecular result. The proband’s parents were healthy regarding symptoms related to HypoPP until their deaths. The arrow represents our proband. His 33-year-old son (IV-6) was heterozygous for this pathogenic variant and affected (black symbol). The slash symbol denotes deceased individuals.

The proband III-5 on the pedigree in Figure 1 was born in Mexico City, Mexico; there is a single case in the family, and he has two sons aged 33 and 26 years. His 33-year-old son had similar symptoms for three years. The onset of symptoms in the proband occurred when he was 11 years old.

One day after swimming, he presented immobility in both pelvic limbs associated with consumption of carbohydrates that triggered muscle weakness in the lower extremities and later in both arms, periodically. Around the same age, he suffered a fall from his bicycle, referring to muscle weakness in his lower limbs moments before the fall. His muscle weakness lasted for approximately three days. At age 23, during one of these episodes, he was diagnosed with hypokalemia requiring administration of potassium orally up to 100 mEq or intravenous, and his symptoms resolved in one or two hours. Previously at age of 22, he presented myalgias of moderate to severe intensity in the left quadriceps, denoting a pattern of asymmetry. He has not been able to climb stairs for six years, and his condition mainly affects his gluteus, biceps, and gastrocnemius muscles. For one year and six months, he noticed improvement when taking beclomethasone twice daily, and he did not require potassium supplementation for at least six months. A muscle biopsy in August 2020 revealed a vacuolar myopathy, the same as what was found in his muscle biopsy performed at ages 13 and 28. We clinically suspect a HypoPP possibly associated with a myofibrillar myopathy-the reason for which we requested a molecular analysis by sequencing and MLPA (multiplex ligation-dependent probe amplification) with a panel of 201 genes associated with neuromuscular diseases.

Genomic DNA obtained from the submitted sample was enriched for targeted regions using a hybridization-based protocol and sequenced using Illumina technology. Reads were aligned to a reference sequence (GRCh37), and sequence changes were identified and interpreted in the context of a single clinically relevant transcript. Confirmation technologies included any of the following: Sanger sequencing, Pacific Biosciences SMRT sequencing, MLPA, MLPA-seq, array CGH. and array CGH confirmation of NGS CNV calling performed by Invitae Corporation (1400 16th Street, San Francisco, CA 94103, #05D2040778). If a rare SNP or indel variant is identified by this method, the gene involved is amplified by long-range PCR and the location of the variant is determined by Pacific Biosciences (PacBio) SMRT sequencing of the relevant exon in both long-range amplicons. If a CNV is identified, MLPA or MLPA-seq is run to confirm the variant.

Molecular testing in the patient identified a missense variant: c.3716G>A (p.Arg1239His) in the *CACNA1S* gene in a heterozygous state, associated with autosomal dominant hypokalemic periodic paralysis 1 (HypoPP-1); autosomal dominant malignant hyperthermia susceptibility (MHS), an autosomal dominant and recessive congenital myopathy. This variant was also detected in his 33-year-old son with similar symptoms concluding the same clinical and molecular diagnosis as his father. The proband’s asymptomatic son of 26 years old was negative for the pathogenic variant in the *CACNA1S* gene. These pathogenic variants in *CACNA1S* cause a loss of the positively charged arginine into other amino acids (histidine, glutamine, serine, glycine, or cysteine in order of frequency) [4].

## Discussion

The *CACNA1S* gene is associated with HypoPP-1 by altering the S4 segment of domains II, III, and IV, differing from the hyperkalemic form due to a spontaneous attack. The gene is associated with hypokalemia, and carbohydrate or sodium-rich food triggers an attack. These are longer and more severe, causing weakness in patients during the day or after strenuous physical activity with all the different clinical presentations of *CACNA1S* following an autosomal dominant mode of transmission. Under some conditions, muscle filaments with severe involvement of a voltage sensor could be depolarized during hypokalemia and at normokalemic episodes; this membrane loss explains episodes of weakness and interictal weakness. Weakness associated with adipose replacement myopathy is very frequently found in patients harboring DIV mutations (fourth domain of the tetrameric structure of the α-subunit of the voltage-gated calcium channel) in the calcium channel (i.e., Cav1.1 p.Arg1239His) [5]. This sequence change was detected in our patient and his son (III-5 and IV-6, respectively) and involved codon 1239 of the CACNA1S protein (p.Arg1239His) involving a highly conserved site and reported in many patients with HypoPP diagnosis [6-11]. This variant is ID number 17623 in ClinVar [12]. Penetrance is approximately 90% in males, but it could be as low as 50% in females [13-15]. Unfortunately, we were unable to access a blood sample from the proband’s parents because they died many years ago this molecular study was performed apparently from unrelated causes to the molecular diagnosis established.

Our patient began at an early age with HypoPP, and later in adulthood, late-onset girdle myopathy was added, and the muscle biopsy presented a rare finding previously reported: the presence of vacuoles [2]. The proportion of HypoPP attributed to pathogenic variants in the *CACNA1S* gene is approximately 40% to 60%, with 7% to 14% in the SCN4A gene. In approximately 30%, the cause is unknown [15]. In vitro studies of this missense change have demonstrated the anomalous gating pore current by altering the permeability of the CACNA1S ion channel [16]. The clinical HypoPP phenotype is indistinguishable from pathogenic variants in *CACNA1S* or *SCN4A* [15,17].

Pathogenic missense variants in the *CACNA1S* that segregated with the MHS phenotype comprised an interesting finding in our patient that developed this clinical spectrum with the variant p.Arg1239His, previously not associated with MHS [18,19]. In a study published by Jurkat-Rott et al. of 36 patients with HypoPP, 28 harbored a pathogenic mutation in the *CACNA1S* gene: p.Arg528His (n=13), p.Arg528Gly (n=1), p.Arg1239His (n=12), and p.Arg1239Gly (n=2) [16]. On the other hand, eight patients had a molecular diagnosis of a pathogenic variant in the SCN4A gene. The p.Arg1239His variant showed the most frequent muscle weakness episodes, and some of the patients had wheelchair dependence, indicating this change was associated with a more severe HypoPP phenotype [17].

Typically, a muscle biopsy is not performed during the approach for HypoPP, and associated histologic findings may depend on the specific pathogenic variant. Because the finding of vacuoles in the muscle biopsy of our patient related to the p.Arg1239His variant in *CACNA1S* had not been previously reported as a known histopathologic feature, our case is particularly useful in the expansion of the phenotype associated with HypoPP-1. Based on our patient’s medical history, it was the reason we suspected channelopathy as the main cause of his symptoms. Since there are previous reports of vacuolar myopathy associated with other genetic causes as part of the differential diagnosis, for example, defects of a lysosomal pathway like a glycogen storage disease II or Pompe disease, Danon disease by a mutation in the LAMP2, in an unusual facioscapulohumeral muscular dystrophy (FSHD) phenotype of X-linked myopathy with excessive autophagy (MEAX) caused by a mutation in the VMA21 gene, an essential assembly chaperone of the vacuolar ATPase [20], we decided to proceed with an extended panel of genes related to these neuromuscular diseases including but not limited to muscular dystrophies, inherited myopathies, mitochondrial disorders, congenital myasthenic syndromes, and rhabdomyolysis. The genetic heterogeneity associated with these conditions can make it difficult to use phenotype as the sole criterion to select a definitive cause. Given the clinical overlap of hereditary neuromuscular disorders, broad panel testing allowed us for an efficient evaluation of many potential genes based on a single clinical indication.

Finally, we assessed a panel of 201 of our patient’s genes related to neuromuscular conditions, and we identified additional variants of uncertain significance in the PRX gene (c.3186G>T [p.Lys1062Asn]); in the RETREG1 gene ([c.17C>T [p.Pro6Leu]), and the SPG11 gene (c.5650C>T [p.Arg1884Cys]), in a heterozygous state in each one. PRX, RETREG1, and SPG11 genes are associated with autosomal recessive Charcot-Marie-Tooth disease type 4F, autosomal recessive hereditary sensory and autonomic neuropathy type 2B, and autosomal recessive hereditary spastic paraplegia 11 (SPG11), juvenile amyotrophic lateral sclerosis 5 and Charcot-Marie-Tooth disease type 2X, respectively. Therefore, these entities did not correlate clinically with our case because of their inheritance pattern. It is important to mention that the absence of detection of different variants does not rule out other vacuolar myopathies, since the analysis carried out was through a panel with 201 genes related to neuromuscular diseases and not by other techniques such as an exome or whole-genome sequencing. This analysis could not be performed on his siblings because they expressed the desire not to know their molecular status.

## Conclusions

The presence of vacuoles in the muscle biopsy in our patient was an unusual finding. Our case confirms that myopathy could progress independently of episodes of muscle weakness, and histopathological findings in muscle biopsy may develop despite the absence of persistent weakness. Because HypoPP is an extremely rare condition, it should be considered part of the differential diagnosis in a patient with symptoms of acute crises of muscle weakness or nonspecific myopathy.

Molecular genetic analyses are an important diagnostic tool in the clinical approach to neuromuscular diseases because these are difficult to diagnose by routine tests and biopsy. Hence genetic analysis is mandatory to diagnose and assess prognosis. These analyses indicate that a specific mutation in any gene can manifest various clinical phenotypes, such as that seen in pathogenic variants in the CACNA1S gene, and in the future, this could be a guide to novel treatments as well as promote more research in this field.
